# Consensus process to agree upon surgical quality assurance processes within a pragmatic, multicentre randomised clinical trial comparing targeted axillary dissection and axillary node clearance: the TADPOLE-TOGETHER project

**DOI:** 10.1136/bmjopen-2024-095774

**Published:** 2025-06-03

**Authors:** Shelley Potter, Ruth Mullan, Henry Cain, Edward St John, Peter Barry, Yazan Masannat, James R Harvey, Katherine Fairhurst, Adrienne Morgan, Margaret Perkins, G Bruce Mann, Jocelyn Lippey, Natalie S Blencowe, Stuart A McIntosh, on behalf of the TADPOLE Trial Management Group, Kerry Avery

**Affiliations:** 1 Translational Health Sciences, Bristol Medical School, Bristol, UK; 2 Bristol Breast Care Centre, NHS North Bristol NHS Trust, Bristol, UK; 3 Patrick G Johnston Centre for Cancer Research, Queen’s University Belfast Faculty of Medicine Health and Life Sciences, Belfast, UK; 4 Breast Surgery, Newcastle Upon Tyne Hospitals NHS Foundation Trust, Newcastle Upon Tyne, UK; 5 Breast Surgery, Portsmouth Hospitals University NHS Trust, Portsmouth, UK; 6 Research Group in Breast Health, University of Portsmouth, Portsmouth, Hampshire, UK; 7 Breast Surgery, Royal Marsden Hospital NHS Trust, London, UK; 8 Mid and South Essex NHS Foundation Trust, Basildon, UK; 9 Nightingale Centre, Wythenshawe Hospital, Manchester, UK; 10 Translational Health Sciences, University of Bristol Medical School, Bristol, UK; 11 ICPV, Independent Cancer Patients’ Voice, London, UK; 12 Breast Surgery, The Royal Melbourne Hospital, Parkville, Victoria, Australia; 13 The Royal Women’s Hospital, Parkville, Victoria, Australia; 14 ANZ Breast Cancer Trials Group Ltd, Hunter Region Mail Centre, New South Wales, Australia; 15 Department of Surgery, University of Melbourne VCCC, Parkville, Victoria, Australia; 16 St Vincent’s BreastScreen, St Vincent’s Hospital (Melbourne) Limited, Fitzroy, Victoria, Australia; 17 Department of Surgery, St Vincent’s Hospital (Melbourne) Limited, Fitzroy, Victoria, Australia; 18 Centre for Surgical Research, School of Social and Community Medicine, University of Bristol, Bristol, UK; 19 Nationwide, Nationwide, UK

**Keywords:** Breast surgery, Breast tumours, Randomized Controlled Trial, Delphi Technique

## Abstract

**Abstract:**

**Introduction:**

Patients with node-positive breast cancer having primary surgery currently undergo axillary node clearance (ANC) to reduce the risk of breast cancer recurrence. Evidence that this highly morbid procedure improves survival is lacking, but approximately 30% of patients will develop lifelong complications which significantly impact their quality of life.

Targeted axillary dissection (TAD) may be a safe, less morbid alternative to ANC and will be evaluated in the upcoming Targeted Axillary Dissection versus axillary node clearance in patients with POsitive axillary Lymph nodes in Early breast cancer (TADPOLE) randomised controlled trial.

TAD is not currently routine practice in patients having primary surgery, so it is vital that the procedure is performed in an agreed upon, standardised way within the trial and procedure fidelity monitored to ensure the results are generalisable and will be accepted by the surgical community. Robust surgical quality assurance (SQA) is essential. Here we describe the first phase of the TADPOLE SQA, a consensus process with the breast surgical community to agree upon how (1) surgery should be performed and standardised; (2) procedure fidelity will be monitored and (3) requirements for surgeon credentialling within the trial.

**Methods and analysis:**

The consensus process will have three phases:

1. Generation of a long list of possible components of TAD from a scoping review and expert opinion. Identified items will be categorised and formatted into Delphi consensus questionnaire items.
2. At least two rounds of an online Delphi survey in which at least 100 breast cancer surgeons will rate the importance of mandating/prohibiting, standardising and/or monitoring each component.
3. A consensus meeting with surgeons to discuss, agree upon and ratify the approach to SQA within TADPOLE.

**Ethics and dissemination:**

Ethical approval has been obtained from the University of Bristol Faculty of Health Sciences Ethics Committee. Educational materials including videos and webinars will be developed and shared with surgeons participating in TADPOLE. Results will be presented at national/international meetings and published in peer-reviewed journals.

**STRENGTHS AND LIMITATIONS OF THIS STUDY**

- Robust consensus methods will be used to agree upon how targeted axillary dissection (TAD) should be performed in patients having primary surgery prior to evaluation in the TADPOLE randomised controlled trial.
- Recommendations for primary TAD and surgical quality assurance within TADPOLE will be developed in partnership with the breast surgical community promoting ownership of the technique and engagement with the TADPOLE trial.
- Development of training and education materials including webinars and videos supported by a network of ‘TAD champions’ will support dissemination to surgeons participating in TADPOLE and promote the standardised introduction and evaluation of TAD.
- Careful surgical quality assurance monitoring of TAD and axillary clearance based on the consensus criteria will be essential to monitor procedure fidelity within the trial.

## Introduction

 In the UK, the National Institute of Health and Care Excellence currently recommends that all patients with node-positive breast cancer having primary surgery undergo an axillary node clearance (ANC),^1^ a radical operation to remove all lymph nodes in the axilla. This highly morbid procedure is recommended to reduce the risk of breast cancer recurrence and improve survival even if patients have only one or two radiologically detected positive nodes: so-called ‘low volume’ nodal disease. There is, however, no evidence that ANC improves breast cancer survival in this group,^2^,^11^ but one in three patients will experience life-changing, lifelong complications such as lymphoedema (20%),^12^ chronic pain (20%)^13^ or problems with shoulder function that will significantly impact their long-term quality of life.

Targeted axillary dissection (TAD) is a newer surgical technique in which only the involved node(s) and the first draining (sentinel) node(s) are removed.^14^ It is less invasive than ANC and may result in fewer complications.^15^ TAD is already routinely performed in patients with node-positive breast cancer who have a complete response to pre-surgical (neoadjuvant) chemotherapy^16^ and may also be a better option for patients with low volume axillary disease having primary surgery as it could reduce the risk of life-changing complications without compromising breast cancer outcomes. However, high-quality evidence from robust clinical trials will be required to support the introduction of TAD in this setting.

The upcoming Targeted Axillary Dissection versus axillary node clearance in patients with POsitive axillary Lymph nodes in Early breast cancer (TADPOLE) study is a large-scale multicentre, pragmatic, phase III randomised controlled trial that aims to compare the clinical and cost-effectiveness of TAD and ANC in patients with low volume node-positive breast cancer having primary surgery. It will test the hypothesis that TAD is superior to ANC in terms of reducing rates of lymphoedema 12 months following surgery without leading to unacceptable rates of locoregional recurrence at 5 years. TAD in patients having primary surgery (termed ‘primary TAD’), however, is not currently routine surgical practice.^17^ Work is therefore required before the trial can commence to agree what components constitute a ‘primary TAD’ procedure and how both procedures should be performed in the future trial.

Surgical quality assurance (SQA) is an essential part of surgical trials.^18^ It is important to ensure that the procedure is being performed in a sufficiently standardised way for the intervention (ie, TAD) and comparator (ie, ANC) to be distinct from each other, enabling a fair comparison, and to allow successful interventions to be replicated in routine practice. Quality assurance processes should consider three areas: (1) standardisation of the intervention, (2) performance monitoring to ensure the allocated procedure is performed as intended (ie, adherence, fidelity, compliance) and (3) credentialling (ie, the level of expertise required for surgeons/centres to participate in the trial).^18^,^20^ Reporting of SQA is integral to the Consolidated Standards of Reporting Trials (CONSORT) extension for non-pharmacological treatments,^21^ but lack of appropriate quality assurance has been a major criticism of surgical oncology trials because it can create difficulties in the accurate interpretation of results and when synthesising findings across trials.^19^ Approaches to SQA within individual trials, however, require flexibility and it is vital that they are tailored depending on the complexity and/or stability of the intervention(s) being evaluated and the degree of pragmatism in the trial design. For TADPOLE, for example, although surgeons are already familiar with TAD in the neoadjuvant setting,^16^ our feasibility work demonstrated few routinely perform TAD in patients with node-positive disease having primary surgery (‘primary TAD’) and highlighted a lack of agreement as to how ‘primary TAD’ should be performed.^17^

The first phase of the TADPOLE SQA (TADPOLE-TOGETHER), therefore, will use consensus methods with the breast surgical community to develop and agree upon the quality assurance strategy for the future trial. This will include agreement on how axillary surgery should be performed, standardised and monitored within the trial. It is anticipated that this collaborative approach will promote ownership of the primary TAD technique and allow it to be introduced and evaluated in a robust, transparent and efficient way within the TADPOLE trial.

**Specific objectives** will be to agree upon

1. The mandatory/prohibited components of TAD and ANC within the trial; the degree of standardisation required and any permitted flexibility in how they are delivered.
2. How procedure fidelity will be monitored including the data to be collected in the case report forms.
3. Credentialling—the level of experience/expertise necessary for surgeons and centres to participate in the trial.

## Methods and analysis

### Overview

TADPOLE-TOGETHER will use a consensus process with the breast surgical community in partnership with ‘TAD Champions’ to develop and agree upon the TADPOLE SQA strategy followed by the development of educational resources to support surgeons participating in the trial.

The consensus process will have three phases:

1. Generation of a comprehensive list of axillary procedure components and concomitant (ie, co-)interventions for the Delphi survey.
2. Prioritisation of identified items using an online Delphi Survey with expert breast surgeons.
3. A consensus workshop/meeting to discuss, agree upon and ratify the SQA processes.

The project commenced in August 2024. The consensus process and development of educational resources are planned to end in Summer 2025 to support the opening of the TADPOLE trial. SQA will continue throughout the recruitment phase of TADPOLE, which is planned to continue until Summer 2027.

### Patient and public involvement

Patient and public involvement is integral to the design of the TADPOLE study. All aspects of the TADPOLE-TOGETHER project will be discussed and reviewed by the TADPOLE Trial Management Group which includes two patient advocates (AM and MP). The agreed upon approach to SQA will be presented to and discussed with the wider TADPOLE Patient Advisory Group (PAG).

### Phase 1: generation of a comprehensive list of procedure components and concomitant interventions

#### Scoping review

A scoping review of the introduction of TAD in the neoadjuvant setting will be undertaken to inform the development of the long list. This approach was chosen as initial scoping work highlighted an absolute paucity of studies reporting TAD performed in the primary surgical setting. TAD in the neoadjuvant setting is now comparatively well established and it was hypothesised that steps and components of the procedure would be broadly applicable to primary TAD. The neoadjuvant TAD literature would therefore have utility and value for identifying items for inclusion in the Delphi survey.

##### Search strategy and data sources

A comprehensive search of online databases MEDLINE and EMBASE will be undertaken to identify primary research studies, published in full reporting the introduction and evaluation of TAD performed in patients following completion of neoadjuvant systemic therapy (NST). The search strategy will be developed in collaboration with a specialist subject librarian and will be limited to human studies, published in English. Search terms will include (“tailored axillary surgery” OR “targeted axillary dissection” OR “selected surgical localisation” OR “targeted axillary sampling” OR “targeted axillary surgery” OR “MARI” OR “RISAS”) AND (“neoadjuvant”). Snowball searching will be used to identify any additional relevant papers from reference lists of included papers and identified review articles.

##### Inclusion and exclusion criteria

All primary research studies published in full, in English, reporting the introduction and/or evaluation of TAD performed in patients who have received NST will be eligible for inclusion. Excluded will be abstracts and conference proceedings due to difficulties interpreting incomplete information, editorials, opinion pieces, reviews and letters. Snowball searching of reference lists of reviews and relevant papers will be used to identify any additional potentially relevant publications.

Abstracts will be imported into EndNote reference management software and screened for inclusion using pre-specified inclusion criteria. Where uncertainty exists, the full text will be obtained for review. Any continued uncertainties will be resolved by discussion with the wider review team.

##### Data extraction

Data will be extracted using a Microsoft Excel data collection proforma iteratively developed and refined by the review team. Approximately 10% of studies will be double data extracted to ensure consistency and methodological rigour. Data extracted will include study details, specifically author and year of publication, study design and number of participants. All details about how the TAD procedure was performed including details of relevant concomitant interventions (eg, if and how the involved lymph node was marked) together with any information regarding surgeon credentialling, procedure standardisation and monitoring within the study were extracted verbatim. Uncertainties will be discussed and resolved by discussion with the wider review team.

##### Analysis

Extracted data will be reviewed and categorised using a content analysis approach.^22^ Steps of the TAD procedure will be classified using an established typology.^23^ The proposed classifications will be reviewed with the wider study team to ensure they are clinically meaningful.

### Expert input from ‘TAD Champions’

An expert group of surgeons with experience of performing primary TAD and/or introduction of novel surgical techniques will be recruited to act as ‘TAD champions’. Individual views will be independently elicited regarding what steps/components/concomitant interventions related to the procedure should be considered for inclusion in the Delphi survey. Champions will also review and comment on the findings of the scoping review as they emerge.

Findings from the scoping review and iterative expert input will be combined to generate a comprehensive long list of all aspects of the procedure and relevant co-interventions that may be subject to variation for inclusion in the Delphi survey (phase 2).

### Phase 2: prioritisation of procedure components and concomitant/co-interventions for standardisation using a stakeholder Delphi survey

A consensus process comprising two sequential rounds of an online Delphi survey followed by a face-to-face consensus meeting will be used to determine the mandatory/prohibited components of TAD/ANC and which components and concomitant interventions should be standardised and/or monitored within the TADPOLE trial. The Delphi process will allow diverse stakeholders from a broad geographic area to participate anonymously and avoid the impact of any dominant groups or individuals.^24^

#### Development of the Delphi survey questionnaire

Each component/concomitant intervention from the long list described above will be operationalised and formatted into a survey item. Identified component will be scored as ‘mandatory’ (ie, MUST be performed in ALL cases of TAD/ANC procedure in the TADPOLE trial); ‘optional’ (ie, up to individual surgeons whether this step should be done/not done as part of the procedure) or ‘prohibited’ (ie, MUST NOT be performed in patients randomised to the procedure). A Likert scale ranging from 1 (not essential) to 9 (absolutely essential) based on the Grading of Recommendations Assessment, Development and Evaluation scale will then be used to determine which component/concomitant intervention should be standardised and/or monitored in the TADPOLE trial.^25^ A list of the ways each component/concomitant intervention could be standardised will be provided based on the findings from phase 1 and respondents stating that a component/concomitant intervention should be standardised will be asked to indicate the one they consider to be the most appropriate. The draft survey will be reviewed by the ‘TAD champions’ and piloted with a small group of breast surgeons prior to launch to ensure face and content validity.

#### Participant sampling and invitations

Consultant, attending or equivalent level breast surgeons and senior trainees (defined as those with a Certificate of Completion of Training (CCT) or within 12 months of their CCT date or equivalent) will be invited to participate in two rounds of an online Delphi survey via the professional associations, social media and professional networks.

#### Delphi survey rounds

Participants will complete two sequential questionnaire rounds over approximately 3 months. In both rounds, each participant will be asked to score each procedure component as mandatory/optional/prohibited before rating the importance of standardising and/or monitoring each procedure component/concomitant intervention in the trial on a 9-point Likert scale from 1 (not essential) to 9 (absolutely essential). All survey questionnaires will be administered using online software to optimise participation.

All participants who complete round 1 of the survey will be sent the round 2 questionnaire. The round 2 survey will include all items from round 1 (see below) together with anonymised feedback in the form of summary scores from round 1 (eg, medians or means). Respondents will then be asked to re-prioritise items in light of the feedback received to promote consensus.^24 26^

Items retained after the round 2 Delphi survey will be carried forward for discussion at the consensus meeting.

#### Attrition between rounds

Participant attrition between rounds will be monitored and differences between those who do/do not complete all survey rounds explored.

### Phase 3: consensus meeting/workshop

A face-to-face consensus meeting attended by expert breast surgeons facilitated by an independent chair will be held to discuss and agree upon the approach to SQA in the TADPOLE trial.

A purposive sample of 20–25 breast surgeons completing both rounds of the Delphi survey will be invited to attend the in-person consensus workshop at which the final approach to standardising and monitoring axillary surgery within the TADPOLE trial will be discussed and agreed upon. Sampling will be based on experience with the techniques and geography to ensure broad representation of the breast surgical community.

The consensus meeting will include a brief presentation outlining the study and the aims of the meeting and presentation survey. Participants will be asked to ratify consensus in/consensus out items and anonymously vote on items for which consensus was not achieved. Rounds of discussion and voting will continue until consensus is achieved. The final approach to surgical quality assurance within the TADPOLE trial will then be ratified by the group.

### Sample size

There is no standard sample size for consensus processes, so the aim will be to ensure good representation from breast surgeons across the UK. It is anticipated that 100–150 surgeons will be involved in the Delphi survey and 20–25 will participate in the consensus meeting.

### Data analysis

#### Survey analysis and provision of feedback

Data will be captured and stored using REDCap software^27^ and downloaded into statistical software for cleaning and analysis.

Respondent demographics for each round will be summarised using descriptive statistics and tabulated. Summary statistics will be calculated for each item and the proportion of participants scoring each item as ‘not essential’ (score 1–3); ‘equivocal’ (score 4–6) and ‘essential’ (score 7–9) calculated. All items will be retained between rounds 1 and 2 so that respondents are able to re-prioritise each item based on the feedback received.

At the end of round 2, items will be categorised as ‘consensus in’, ‘consensus out’ or ‘no consensus’ as described in table 1.

**Table 1:** Definitions of consensus (following survey round 2)

Category	Score	Outcome
‘Consensus in’	Components rated as ‘mandatory’ OR ‘prohibited’ by ≥70%Standardisation/monitoring items scored as essential (7–9) by ≥70% and not essential (1–3) by <15%Method of standardisation agreed upon by ≥70%	Discussed/ratified at consensus meeting
‘Consensus out’	Standardisation/monitoring items scored as not essential (1–3) by ≥70% and essential (7–9) by <15%	Discussed/ratified at consensus meeting
‘No consensus’	None of the criteria above are met	Discussed and voted on at consensus meeting

#### Consensus meeting

Following voting, items will be categorised as ‘consensus in’ according to the definition in table 1 and included in the SQA guidelines. Items not reaching this threshold will be considered ‘consensus out’ and discarded. A final vote will be used to ratify the approach to SQA to be used within the TADPOLE trial.

### Development of educational/training resources for surgeons participating in the TADPOLE trial

Educational materials including videos and webinars will be developed based on the consensus standardisation guidelines for the performance of TAD within the TADPOLE trial in collaboration with the trial TAD Champions (HC/PB/JH/ESJ) and the TADPOLE Educational Lead (YM). These resources will be widely promoted and disseminated in conjunction with the relevant professional associations and will support surgeons participating in the trial. The standardisation guidelines will be set out in the TADPOLE surgical manual which will accompany the protocol, and guidelines incorporated into TADPOLE Site Initiation Visits. During the trial, virtual site clinics will be held with TAD Champions to offer further support to participating surgeons and teams. This will ensure that primary TAD is introduced and implemented within in a standardised way within the trial and beyond.

## Ethics and dissemination

Ethical approval for the project will be obtained from the University of Bristol Faculty of Health Sciences Research Ethics committee (reference 20773). Participation in the Delphi survey by professional participants will be taken as implied consent. Formal written consent will be obtained from surgeons prior to their participation in the consensus meeting.

Findings will be presented at international meetings and published in peer-reviewed journals. Dissemination materials will be produced in collaboration with the TADPOLE TMG and PAG so the results can be shared widely.

Most importantly, the findings of this study will be used to develop educational materials for surgeons participating in the TADPOLE trial. These will be shared widely within the breast surgical community via the professional associations, study website and other social media channels to ensure that primary TAD is introduced in a robust and standardised way prior to definitive evaluation in the TADPOLE trial. This, in combination with effective monitoring of procedure fidelity, will ensure that the trial results are credible and meaningful, and if positive, TADPOLE will provide practice-changing evidence for the safe and rapid implementation of the primary TAD, reducing the morbidity associated with axillary surgery in node-positive breast cancer and improving outcomes for patients.
