# Supplementary material for: Consensus process to agree upon surgical quality assurance processes within a pragmatic, multicentre randomised clinical trial comparing targeted axillary dissection and axillary node clearance: the TADPOLE-TOGETHER project
Source: BMJ Open. 2025 Jun 3;15(6):e095774. doi: 10.1136/bmjopen-2024-095774 (PMC12142125; doi:10.1136/bmjopen-2024-095774)
Supplement: Supplementary file 1 [file bmjopen-2024-095774.reviewer_comments.pdf]

PEER REVIEW HISTORY

BMJ Open publishes all reviews undertaken for accepted manuscripts. Reviewers are asked to complete a checklist review form and are provided with free text boxes to elaborate on their assessment. These free text comments are reproduced below.

ARTICLE DETAILS

Title (Provisional)

A consensus process to agree upon surgical quality assurance processes within a pragmatic, multicentre randomised clinical trial comparing targeted axillary dissection and axillary node clearance: The TADPOLE-TOGETHER project

Authors

Potter, Shelley; Mullan, Ruth; Cain, Henry; John, Edward St; Barry, Peter; Masannat, Yazan; Harvey, James R; Fairhurst, Katherine; Morgan, Adrienne; Perkins, Margaret; Mann, G. Bruce; Lippey, Jocelyn; Blencowe, Natalie S; McIntosh, Stuart; TADPOLE Trial Management Group, The

---

VERSION 1 - REVIEW

**Reviewer**	**1**
**Name**	**Weiss, Anna**
**Affiliation**	**University of Rochester**
**Date**	**29-Nov-2024**
**COI**	**None**

This is a really well written manuscript, and important work. I have just a few small comments.

Throughout the manuscript "agree" is used and should likely read "agree upon" or "agreed upon". Please change throughout.

In the introduction the 3 objectives are listed in a different order than when the specific objectives are listed (i.e. credentialing is listed first in the earlier mention, but last in the specific objectives). Please keep consistent.

Will the authors be submitting a similar manuscript for the TADPOLE trial itself? I am pleased to see this initial QA step submitted, but a plan for the trial itself will greatly add to the literature.

**Reviewer**	**2**
**Name**	**Kataria, Kamal**

**Affiliation**	**All India Institute of Medical Sciences New Delhi, Surgical Disciplines**
**Date**	**20-Mar-2025**
**COI**	**None**

It is a well designed protocol. Best wishes

VERSION 1 - AUTHOR RESPONSE

Reviewer: 1 Comments: Dr. Anna Weiss, University of Rochester

1. ***This is a really well written manuscript, and important work. I have just a few small comments. Throughout the manuscript "agree" is used and should likely read "agree upon" or "agreed upon". Please change throughout.***

   Thank you for highlighting this. It has been corrected throughout the manuscript.

2. ***In the introduction the 3 objectives are listed in a different order than when the specific objectives are listed (i.e. credentialing is listed first in the earlier mention, but last in the specific objectives). Please keep consistent.***

   We agree consistency is important so have revised the order of the objectives in the introduction so that they reflect the specific objectives listed.

3. ***Will the authors be submitting a similar manuscript for the TADPOLE trial itself? I am pleased to see this initial QA step submitted, but a plan for the trial itself will greatly add to the literature.***

   We thank Dr Weiss for this question. The TADPOLE trial is currently undergoing regulatory review and ethical approval. Once these approvals are in place, we will aim to submit a similar protocol paper for the main TADPOLE trial. We anticipate this will be Summer 2025.

Reviewer: 2 Comments: Dr. Kamal Kataria, All India Institute of Medical Sciences New Delhi

1. ***It is a well designed protocol***.

   Thank you.
